# The effect of maltobionic acid on bone metabolism markers in healthy Japanese postmenopausal women: A randomized double‐blind placebo‐controlled crossover study

**DOI:** 10.1002/fsn3.2855

**Published:** 2022-04-01

**Authors:** Daiki Suehiro, Yuichiro Moriwaki, Ken Fukami, Sumiko Abe‐Dohmae, Motoko Ohnishi

**Affiliations:** ^1^ San‐ei Sucrochemical Co., Ltd. Aichi Japan; ^2^ Graduate School of Bioscience and Biotechnology Chubu University Aichi Japan; ^3^ College of Bioscience and Biotechnology Chubu University Aichi Japan

**Keywords:** bone formation, bone metabolism, bone resorption, maltobionic acid, oligosaccharide

## Abstract

Osteoporosis is characterized by compromised bone strengthpredisposing to an increased risk of fracture and is a disease with a high incidence in postmenopausal women. Frequent estrogen deficiency, particularly in postmenopausal women, induces osteoclast activation and is a major contributor to reduced bone mineral density. Maltobionic acid (MB) reportedly promotes mineral resorption and maintains bone mineral density in human clinical trials, although no studies have confirmed that MB improves bone metabolism in humans. Therefore, this study aimed to investigate the effects of MB administration on bone‐resorption markers in healthy Japanese postmenopausal women. This was a randomized, double‐blind, placebo‐controlled, crossover trial. Twenty‐six healthy adult Japanese women who realized that they had passed through more than 1 year of natural menopause and were aged 40–69 years were categorized into three groups. The experimental groups were allowed to consume maltobionic acid syrup 4 g (MB syrup 4 g group), maltobionic acid syrup 2 g plus maltose syrup 2 g (MB syrup 2 g group), and maltose syrup 4 g (placebo group) for 4 weeks. All 26 participants completed the intervention. Continuous ingestion of MB syrup 2 g or 4 g for 4 weeks significantly reduced the levels of bone‐resorption markers deoxypyridinoline (DPD) and urinary N‐telopeptide (u‐NTx), and significantly increased the bone formation marker osteocalcin (OC) compared with the placebo group. Maltobionic acid (MB) intake may improve bone metabolism and reduce bone health problems, including osteoporosis, in postmenopausal, adult Japanese women. (UMIN‐CTR ID: UMIN000038627).

## Graphical abstract

Maltobionic acid (MB) reportedly promotes mineral resorption and maintains bone mineral density in humans, although its effects on bone metabolism are unknown. This study investigated the effects of MB administration on bone‐resorption and ‐formation markers in healthy, adult, Japanese women through a randomized, double‐blind, placebo‐controlled, crossover trial. Continuous ingestion of MB significantly reduced the levels of bone‐resorption biomarkers and increased that of a bone‐formation biomarker. This has implications for reducing bone health problems, including osteoporosis, in postmenopausal women. MB, maltobionic acid; u‐NTx, urinary N‐telopeptide; OC, osteocalcin.

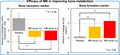

## 1 INTRODUCTION

Osteoporosis has been defined as a skeletal disorder that is characterized by reduced bone strength and an increased risk of fracture (NIH Consensus Development Panel on Osteoporosis Prevention, Diagnosis and Therapy, 2001). Bone, which acts as a reservoir of calcium, continuously cycles through stages in which old bone is lysed and resorbed by osteoclasts (bone resorption), followed by new bone generation by osteoblasts (bone formation) (Parfitt, 1994; de Vernejoul, 1996). An imbalance in bone metabolism results in decreased bone mineral density when bone resorption by osteoclasts exceeds bone formation by osteoblasts, increasing the risk of osteoporosis (Rachner et al., 2011). Frequent estrogen deficiency, particularly in postmenopausal women, induces osteoclast activation and is a major contributor to reduced bone mineral density (Vanderschueren et al., 2014; Zebaze et al., 2010). Daily nutrient intake and nutritional balance greatly affect bone density and metabolism. Calcium is a major inorganic component of bone, and there is a significant relationship between calcium intake and bone metabolism and bone mineral density, and ensuring adequate calcium intake is important for preventing osteoporosis (Nieves et al., 1998). However, the ability to absorb calcium from the gastrointestinal tract decreases with age, in relation to the decreased secretion of gastric acid (Heaney et al., 1989; Nordin et al., 2004). Therefore, the nutritional status of calcium depends not only on intake, but also on how efficiently it can be absorbed within the intestine. The absorption efficiency of calcium is affected by various factors, such as the solubilization state of calcium in the gastrointestinal tract and the food components that are present (Spencer et al., 1966; Sun et al., 2018).

Maltobionic acid (MB) (4‐O‐α‐D‐glucopyranosyl‐D‐gluconic acid: CAS No. 534–42–9) is an indigestible disaccharide with gluconate bound to glucose by α‐1,4 glycosidic bond (Tanabe et al., 2020). A 4‐week clinical trial of MB ingestion among adult women with constipation has confirmed its efficacy in improving bowel movements (Suehiro et al., 2020). In addition, MB is characterized by its ability to form a stable salt with an inorganic cation; therefore, it maintains high water solubility, even when ionically bonded with minerals such as calcium (Kawai et al., 2019). In rodent studies, MB promotes the body retention rates of calcium, magnesium, and iron by maintaining the solubilization state of minerals throughout the gastrointestinal tract (Suehiro, Kawase, et al., 2020; Suehiro et al., 2019). Similarly, in human clinical trials, the oral intake of calcium maltobionate promotes mineral resorption and improves bone mineral density (Fukami et al., 2019). In addition, interventional studies involving calcium maltobionate intake in postmenopausal women have confirmed the suppressive effects of deoxypyridinoline (DPD), a bone‐resorption marker, and type I collagen cross‐linked N‐telopeptide (NTx) (Suehiro et al., 2020). However, it remains unclear whether the intake of free MB, as well as calcium maltobionate, functions effectively for the improvement of bone metabolism. Therefore, this study aimed to investigate the effects of MB ingestion on bone metabolism markers in healthy Japanese postmenopausal women.

## 2 MATERIALS AND METHODS

### 2.1 Study design and subjects

The study was conducted in a randomized, double‐blind, placebo‐controlled, crossover trial. The study participants were publicly recruited, adult Japanese women who realized that they had passed through more than 1 year of natural menopause. All participants were aged 40–69 years and worked at Chubu University (Aichi, Japan). The purpose of the study and its methodology were fully explained to those who wanted to participate. A preliminary questionnaire was administered to those who provided written consent, and those who did not meet the following exclusion criteria were selected: (1) a medical history of treatment for malignancy, cardiac failure, or myocardial infarction; (2) a history of other diseases (arrhythmia, hepatic disorder, cerebrovascular disorder, rheumatism, diabetes mellitus, hypertension, and other chronic diseases); (3) the regular use of pharmaceutical agents (including Chinese herbs); (4) the regular ingestion of Food for Specified Health Uses or Foods with Function Claims; (5) at least once‐weekly consumption of supplements, food for specified health uses, and foods with functional claims of effects on bone metabolism, including calcium, magnesium, vitamin D, vitamin K, and isoflavones (including equol, genistein, daidzein, etc.); and (6) allergies to foods related to the test food used in this trial. Premenopausal subjects were also excluded, as were those experiencing premature menopause related to genetic factors, disease, or previous medical procedures.

The study protocol was approved on October 31, 2019 by the Chubu University Certified Review Board (approval no. 20190080) and was conducted in accordance with the Declaration of Helsinki (2013) and the Ethical Guidelines for Medical and Health Research Involving Human Subjects. This study was registered with the University Hospital Medical Information Network (no. UMIN000038627).

### 2.2 Selection, randomization, and blinding

Of the 45 study participants who agreed to participate in the study, 26 were selected based on the results of a preliminary screening questionnaire. Nine, eight, and nine subjects were assigned to sequences 1, 2, and 3, respectively. The age, body mass index (BMI), and young adult mean (YAM) value of the right calcaneus did not differ significantly among the sequences. Group assignment was performed using an interim test controller with randMS (FileMaker, Inc.). At the time of allocation, the participants in the study, the analysts, and other personnel involved were not informed of the group assignments and were not involved in the group allocations. The YAM value in the right calcaneus was determined using an ultrasonic bone densitometry device (CM‐200, Canon Lifecare Solutions Inc.).

### 2.3 Test food and study design

The test food was MB syrup (containing 40 wt% maltobionic acid, SourOligo, San‐ei Sucrochemical Co., Ltd.). In this study, the concentration of MB syrup was set at 2 g and 4 g per day, with reference to the effective intake concentration of calcium maltobionate with improving effect on bone metabolism, as reported by a previous study (Suehiro, Nishio, et al., 2020). Therefore, the placebo group was given maltose syrup (containing 40 wt% maltose, San‐ei Sucrochemical Co., Ltd.). The following three types of syrup were used in the present study: MB syrup 4 g (maltobionic acid syrup 4 g, equivalent to 1.6 g of maltobionic acid), MB syrup 2 g (maltobionic acid syrup 2 g and maltose syrup 2 g, equivalent to 0.8 g of maltobionic acid), and placebo syrup (maltose syrup 4 g).

Prior to the start of the study, the Institutional Review Board confirmed that the foods could not be distinguished based on their odor or color. The participants ingested one packet (4 g) per day at no fixed time. The study schedules included Intervention Trial 1 (4 weeks), a washout period (2 weeks), Intervention Trial 2 (4 weeks), another washout period (2 weeks), and Intervention Trial 3 (4 weeks). During the Intervention Trial, the subjects were instructed to follow their regular lifestyle habits, including normal diet and exercise habits.

### 2.4 Outcome measures

Examinations were performed a total of six times before and after the intervention.

#### 2.4.1 Blood–bone metabolism marker and serum calcitonin levels

Approximately 150 μl of capillary blood was collected using a fingertip blood sampling kit (MBS Capillary, Micro Blood Science Inc.) 1 day before the start of the intervention and 1 day after its end, and the serum was isolated and collected by centrifugation at 4°C and 1,500 *g* for 5 min. The levels of serum osteocalcin (OC), a bone formation marker, and serum calcitonin, a calcium‐regulating hormone, were measured using a Human Osteocalcin ELISA (enzyme‐linked immunosorbent assay) kit (BioVendor Research and Diagnostic Products) and a Calcitonin Human EIA (enzyme immunometric assay) kit (Phoenix Pharmaceuticals, Inc.), respectively.

#### 2.4.2 Urinary bone metabolism marker

Approximately 10 ml of each participant's first morning urine samples was collected 1 day before the start of the intervention and 1 day after its end. The recovered urine was corrected for urinary creatinine content after measurement of the bone‐resorption markers, DPD and u‐NTx. DPD and u‐NTX levels were measured using ELISA kits (Osteolinks‐DPD; SB Bioscience Co., Ltd.) and OSTEOMARK (Alere Medical Co., Ltd.), respectively. Urinary creatinine was measured using the LabAssay^™^ Creatinine colorimetric method (FUJIFILM Wako Pure Chemical Corporation).

#### 2.4.3 General urine test

Approximately 10 ml of first morning urine samples was collected 1 day before the start of the intervention and 1 day after its end. Protein, glucose, urobilinogen, bilirubin, ketone, pH, and occult blood levels were measured in the urine samples. Uropaper Iii Eiken (Eiken Chemical Co., Ltd.) was used to make the visual assessments.

#### 2.4.4 Nutritional survey

Calcium, potassium, vitamin D, and vitamin K are factors that are known to maintain bone mineral density and bone metabolism (Ha et al., 2020; Mott et al., 2019; Reid, 2017; Tai et al., 2015), and the MB evaluated in this study was expected to have similar effects. To correctly evaluate the effects of the intervention foods, they were assessed before and after the start of the intervention using a validated, self‐administered Brief‐type Diet History Questionnaire (BDHQ) prior to and after the initiation of the intervention (Kobayashi et al., 2011, 2012). The BDHQ surveyed the consumption of 56 types of food and beverages in the previous month, and the energy levels and intake of each dietary factor and selected nutrient (carbohydrates, proteins, lipids, calcium, potassium, vitamin D, and vitamin K) were estimated.

### 2.5 Statistical analysis

Data are expressed as the mean ± standard error (*SE*) values. The results were compared both within and between groups. Within‐group comparisons were performed using the paired Student's *t* test to assess the changes between the pre‐ingestion and 4‐week postingestion measures. Between‐group comparisons were conducted using paired the Student's *t* test to assess the differences in changes at each time point in the MB syrup 2 g group or MB syrup 4 g group compared with those in the placebo group. The amount of change was defined as the 4‐week postingestion value minus the pre‐ingestion value. Pre‐ingestion measures and changes were compared between groups using Student's *t* test, whereas the 4‐week postingestion measures were compared between groups via analysis of covariance (ancova), with the pre‐ingestion measures used as the covariates. All statistical analyses were performed using two‐tailed tests, and the significance level was set at 5%. Bell Curve for Excel (Social Survey Research Information) and Microsoft Excel for Microsoft 365 (Microsoft Japan) were used for the statistical analyses. Multiplicity at other time points and other items were not considered.

## 3 RESULTS

### 3.1 Subject characteristics

**FIGURE 1:**
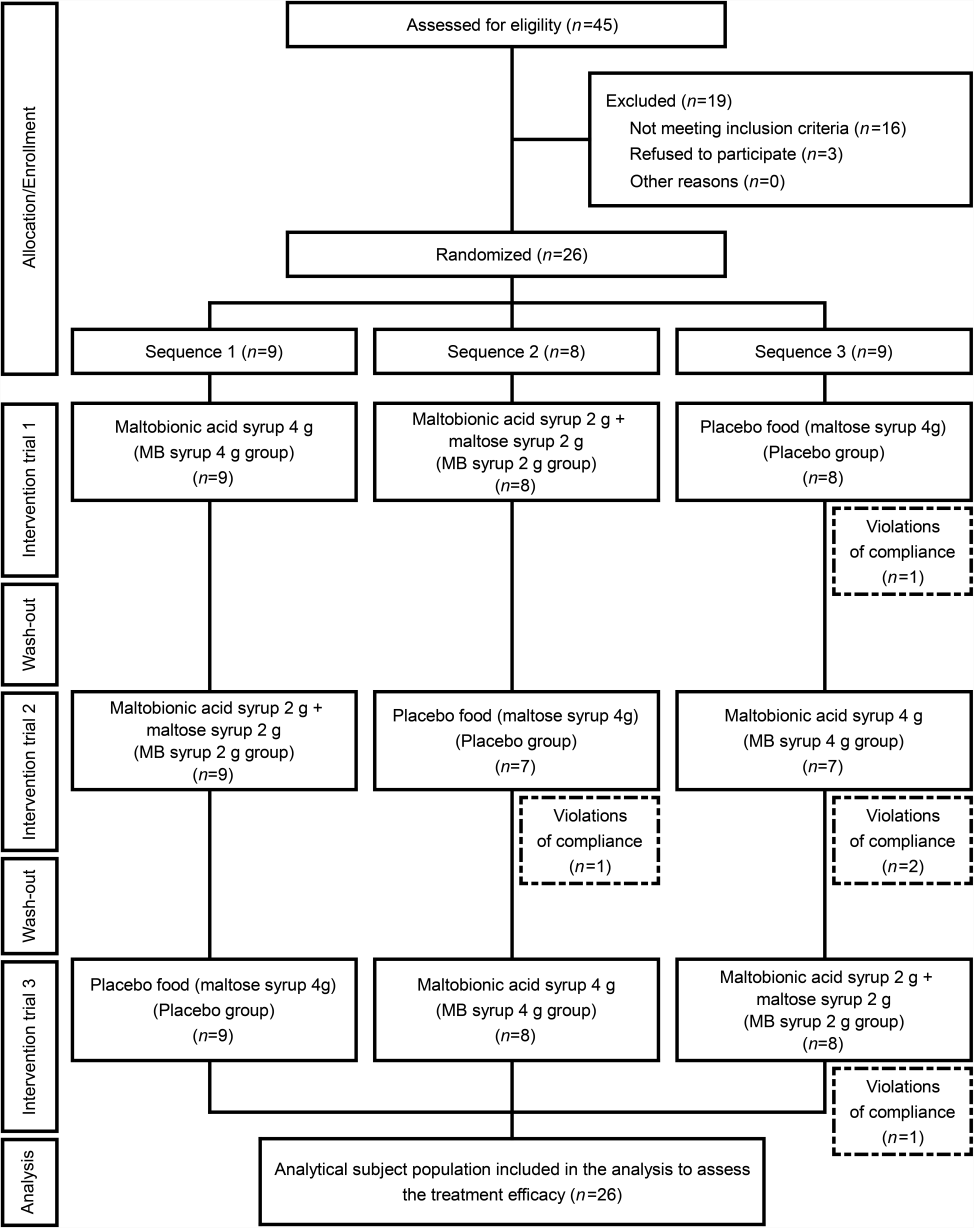
Follow‐up flowchart for the study participants. The trial was performed in three periods using a three‐group, crossover design. The placebo maltose syrup (maltose syrup: 4 g), MB 2 g syrup (maltose syrup: 2 g + maltobionic acid syrup: 2 g), and MB 4 g syrup (maltobionic acid syrup: 4 g) foods were ingested following 2‐week washout intervals

Figure 1 shows the follow‐up flowchart of the study participants. All 26 study participants who agreed to participate and fulfilled the eligibility criteria completed the intervention trial. In a total of three intervention trials, participants who violated any of the compliance items (e.g., an eating rate of test foods ≤80%) and those whose submitted blood samples did not meet the required volume for analysis were excluded from the analysis of each relevant test item. There were no trial participants who did not receive any intervention due to refusal to participate or lack of no postrandomization data. Therefore, the number of subjects included in the efficacy analysis was the same as that in the full analysis set; they comprised a total of 26 subjects (mean age, 56.0 ± 1.0 years), including nine for Sequence 1, eight for Sequence 2, and nine for Sequence 3. We also assessed groups and foods for all outcome data by ancova with generalized linear models by using group and food as fixed factors and each study participant's measurements as variable factors; these analyses revealed no timing, carryover, or ordinal effects. Therefore, the validity of the crossover used in this study was confirmed.

### 3.2 Efficacy assessment

The basic demographic and clinical data of all of the study participants and the 26 subjects included in the analyses are shown in Table 1.

**TABLE 1:** Characteristics of subjects participating in this study

	Overall population (*n* = 45)	Analytical subject population (*n* = 26)
Age (years)	53.0 ± 0.9	56.0 ± 1.0
Body height (cm)	157.5 ± 0.7	156.8 ± 0.9
Body weight (kg)	54.4 ± 1.0	52.7 ± 1.4
BMI (kg/m^2^)	21.9 ± 0.4	21.4 ± 0.5
YAM (%)	78.7 ± 0.5	78.9 ± 0.7

Note

Values represent the mean ± *SE*.

Abbreviations: BMI, body mass index; YAM, young adult mean; *SE*, standard error.

#### 3.2.1 Bone‐metabolism marker and serum calcitonin levels

The results of the efficacy assessment based on DPD, u‐NTx, OC, and serum calcitonin measures are summarized in Table 2. Within‐group comparisons revealed a significant reduction in DPD levels in the MB syrup 4 g group between the pre‐ingestion and 4‐week postingestion time points (*p* = .042). The intergroup comparisons revealed significantly lower values in the MB syrup 2 g group (*p* = .048) and the MB syrup 4 g group (*p* = .042) than in the placebo group at 4 weeks postingestion. In addition, the change from the pre‐ingestion level was significantly lower in the MB syrup 2 g group (*p* = .042) and the MB syrup 4 g group (*p* = .036) than in the placebo group.

**TABLE 2:** Changes in bone metabolism markers (DPD, u‐NTx, OC) and calcitonin

Units	Group	*n*	Pre‐ingestion	Postingestion (4 weeks )	Pre‐ versus. Postingestion (4 weeks)
					Amount of change
DPD (nmol/mmol·Cr)	Placebo	24	5.24 ± 0.30	5.49 ± 0.24	0.25 ± 0.21
	MB syrup 2 g	25	5.21 ± 0.20	4.83 ± 0.16^#^	−0.38 ± 0.19 ^#^
	MB syrup 4 g	24	5.42 ± 0.30	4.69 ± 0.19* ^,^ ^#^	−0.73 ± 0.25 ^#^
u‐NTx (nmol BCE/mmol·Cr)	Placebo	24	52.1 ± 4.8	69.1 ± 9.9	16.9 ± 7.3
	MB syrup 2 g	25	57.5 ± 5.9	49.0 ± 5.7^#^	−8.5 ± 4.6 ^#^
	MB syrup 4 g	24	58.4 ± 7.3	45.2 ± 5.0* ^,^ ^#^	−13.2 ± 7.1 ^#^
OC (ng/ml)	Placebo	16	9.92 ± 1.04	10.12 ± 1.20	0.20 ± 0.48
	MB syrup 2 g	18	9.03 ± 0.83	10.58 ± 0.70	1.55 ± 0.53 ^#^
	MB syrup 4 g	16	9.54 ± 1.10	11.85 ± 0.78* ^,^ ^#^	2.30 ± 0.65 ^#^
Calcitonin (ng/ml)	Placebo	24	6.42 ± 0.27	6.19 ± 0.30	−0.23 ± 0.22
	MB syrup 2 g	25	6.43 ± 0.27	6.93 ± 0.32^#^	0.50 ± 0.21 ^#^
	MB syrup 4 g	24	6.43 ± 0.30	6.50 ± 0.28	0.07 ± 0.18

Note

Values represent the mean ± *SE*. Placebo: maltose syrup (maltose syrup: 4 g), MB 2 g syrup (maltose syrup: 2 g + maltobionic acid syrup: 2 g), and MB 4 g syrup (maltobionic acid syrup: 4 g).

Abbreviations: DPD, deoxypyridinoline; u‐NTx, urinary N‐telopeptide; BCE, bone collagen equivalents; OC, osteocalcin.

* *p* < .05 (versus. Pre‐ingestion);

^#^ *p* < .05 (versus. Placebo).

The within‐group comparisons revealed a significant reduction in u‐NTx levels in the MB syrup 4 g group between the pre‐ingestion and 4‐week postingestion time points (*p* = .043). Intergroup comparisons revealed significantly lower values in the MB syrup 2 g group (*p* = .034) and the MB syrup 4 g group (*p* = .028) compared with the placebo group at the 4‐week postingestion time point. In addition, the change from the pre‐ingestion level was significantly lower in the MB syrup 2 g group (*p* = .023) and the MB syrup 4 g group (*p* < .01) than in the placebo group.

The within‐group comparisons revealed that the serum OC level was significantly increased in the MB syrup 4 g group (*p* = .047) between the pre‐ingestion and 4‐week postinjection time points. Intergroup comparisons demonstrated significantly higher values in the MB syrup 4 g group (*p* = .024) than in the placebo group at the 4‐week postingestion time point. In addition, the change from the pre‐ingestion level was significantly higher in the MB syrup 2 g group (*p* = .017) and the MB syrup 4 g group (*p* < .01) than in the placebo group.

Between‐group comparisons also revealed significant increases in serum calcitonin levels in the MB syrup 2 g group compared with the placebo group at 4 weeks postingestion (*p* = .049) and a greater amount of change from pre‐ingestion to 4 weeks postingestion (*p* = .044).

#### 3.2.2 General urine testing

The results of the general urinalysis are shown in Table 3. In the general urinalysis, false positives and positives were observed sporadically for some measures, although all were transient, and there were no items showing significant differences before and after ingestion or between the groups. Therefore, it was judged that the changes observed did not result in obvious medical problems.

**TABLE 3:** Changes in urinalysis parameters

	Reference value	Group	*n*	Pre‐ingestion	Postingestion (4 weeks)
Protein	(−)	Placebo	24	(−):22, (±):2	(−):21, (±):3
		MB syrup 2 g	25	(−):24, (±):1	(−):24, (±):1
		MB syrup 4 g	24	(−):23, (±):1	(−):23, (±):1
Glucose	(−)	Placebo	24	(−):24	(−):22, (±):1, (+):1
		MB syrup 2 g	25	(−):25	(−):23, (±):2
		MB syrup 4 g	24	(−):23, (+):1	(−):22, (±):2
Urobilinogen	(±)	Placebo	24	(±):24	(±):22, (+):2
		MB syrup 2 g	25	(±):25	(±):23, (+):2
		MB syrup 4 g	24	(±):23, (+):1	(±):24
Bilirubin	(−)	Placebo	24	(−):23, (+):1	(−):23, (+):1
		MB syrup 2 g	25	(−):25	(−):24, (2+):1
		MB syrup 4 g	24	(−):21, (+):3	(−):24
Ketone	(−)	Placebo	24	(−):24	(−):24
		MB syrup 2 g	25	(−):25	(−):25
		MB syrup 4 g	24	(−):24	(−):24
pH	5.0–7.0	Placebo	24	(5.0–7.0):23, (8.0):1	(5.0–7.0):23, (8.0):1
		MB syrup 2 g	25	(5.0–7.0):23, (8.0):2	(5.0–7.0):25
		MB syrup 4 g	24	(5.0–7.5):21, (8.0):3	(5.0–7.5):19, (8.0):5
Occult blood	(−)	Placebo	24	(−):24	(−):24
		MB syrup 2 g	25	(−):25	(−):24, (±):1
		MB syrup 4 g	24	(−):23, (±):1	(−):24

Note

The number of subjects with each result is shown. Placebo: maltose syrup (maltose syrup: 4 g), MB 2 g syrup (maltose syrup: 2 g + maltobionic acid syrup: 2 g), and MB 4 g syrup (maltobionic acid syrup: 4 g).

#### 3.2.3 Nutritional survey

The results of the findings of the BDHQ dietary survey are shown in Table 4. Based on the BDHQ nutritional survey, the intake of nutrients that may affect bone metabolism did not differ significantly either within or between groups.

**TABLE 4:** Nutritional survey (BDHQ)

Item	Units	Ingested food	*n*	Pre‐ingestion	Postingestion (4 weeks)
Calories	kcal/day	Placebo	24	1,714 ± 300	1,732 ± 323
		MB syrup 2 g	25	1,740 ± 308	1,709 ± 300
		MB syrup 4 g	24	1,670 ± 285	1,750 ± 300
Carbohydrate	g/day	Placebo	24	221 ± 49	218 ± 48
		MB syrup 2 g	25	226 ± 56	217 ± 46
		MB syrup 4 g	24	216 ± 58	224 ± 50
Protein	g/day	Placebo	24	69.6 ± 15.1	70.8 ± 15.4
		MB syrup 2 g	25	73.3 ± 18.8	73.0 ± 17.6
		MB syrup 4 g	24	70.3 ± 16.4	72.1 ± 14.7
Fat	g/day	Placebo	24	54.1 ± 13.0	58.5 ± 14.0
		MB syrup 2 g	25	56.8 ± 14.0	56.7 ± 13.7
		MB syrup 4 g	24	53.7 ± 11.7	56.9 ± 14.2
Calcium	mg/day	Placebo	24	547 ± 125	589 ± 150
		MB syrup 2 g	25	570 ± 136	592 ± 167
		MB syrup 4 g	24	559 ± 131	560 ± 133
Potassium	mg/day	Placebo	24	2,662 ± 448	2,820 ± 458
		MB syrup 2 g	25	2,717 ± 505	2,800 ± 487
		MB syrup 4 g	24	2,717 ± 544	2,776 ± 583
Vitamin D	µg/day	Placebo	24	10.8 ± 2.8	11.6 ± 3.3
		MB syrup 2 g	25	11.5 ± 2.5	11.2 ± 3.1
		MB syrup 4 g	24	10.8 ± 3.1	11.3 ± 3.5
Vitamin K	µg/day	Placebo	24	316 ± 100	340 ± 109
		MB syrup 2 g	25	309 ± 96	334 ± 80
		MB syrup 4 g	24	323 ± 91	331 ± 107

Note

Values represent the mean ± *SE*. Placebo: maltose syrup (maltose syrup: 4 g), MB 2 g syrup (maltose syrup: 2 g + maltobionic acid syrup: 2 g), and MB 4 g syrup (maltobionic acid syrup: 4 g).

Abbreviations: BDHQ, brief‐type diet history questionnaire; *SE*, standard error.

## 4 DISCUSSION

The purpose of this study was to investigate the effect of MB intake on bone metabolism in healthy Japanese women aged 40–69 years after menopause for at least 1 year. The bone‐resorption markers DPD and u‐NTx were not altered in the placebo group postingestion in comparison with the pre‐ingestion values, whereas significant reductions in marker levels were observed in the MB syrup 2 g and 4 g groups. Among them, a marked reduction was identified in the change from the pre‐ingestion u‐NTx levels in the MB syrup 2 g group (*p* = .023) and the MB syrup 4 g group (*p* < .01). Furthermore, changes in blood calcitonin levels, measured between the pre‐ and postingestion time points in this study, were significantly increased following ingestion of MB syrup 2 g and slightly increased following ingestion of MB syrup 4 g. Calcitonin is a type of peptide hormone secreted by the parafollicular cells of the thyroid gland that reduces blood calcium level (Wolfe et al., 1974), and its secretion is stimulated by increase in serum calcium concentration (Deftos et al., 1973). Calcitonin inhibits bone resorption by acting directly on osteoclasts, thereby inhibiting the release of calcium from the bone matrix into the blood and lowering blood calcium concentrations by promoting its transfer from the blood to the bone matrix (Gray and Munson, 1969; Yamashita & Hagiwara, 1990). Therefore, MB was supposed to enhance calcitonin secretion which contributed to the suppression of the bone resorption by maintaining the solubilization of calcium contained in daily meals, even if it was ingested in free acid form.

Calcitonin induces the expression of Wnt ligands (such as Wnt10b) in osteoclasts, and it is known that it also promotes osteogenesis by acting indirectly on osteoblasts. (Hsiao et al., 2020). In this study, in comparison with the pre‐ingestion levels of OC, an osteogenic marker, the postingestion changes were significantly higher following the intake of MB syrup at 2 g or more than following the ingestion of placebo. Bisphosphonates, including etidronate and risedronate, are among the most widely used treatments for osteoporosis and bone metabolism disorders (Bilezikian, 2009; Sweet et al., 2009). Pharmacologically, bisphosphonates potently suppress bone turnover, while simultaneously suppressing bone resorption as well as bone formation (Naylor et al., 2016). Therefore, if an extreme decrease in bone formation markers is observed during a period of medication, it is necessary to prescribe an alternative drug or to consider treatment suspension (Camacho et al., 2016). Since the intake of MB did not seem to inhibit bone turnover, it was considered a useful food material with bone metabolism improvement effect that can be continuously consumed.

On the other hand, further studies are required to elucidate the mechanism of the bone metabolism ameliorating effect by ingestion of MB. A mechanism of action for the effect of MB ingestion on improving bone metabolism increased secretion of calcitonin associated with accelerated calcium absorption, but the mechanism of action has not been comprehensively evaluated. For example, soy isoflavones, known to have bone‐resorption inhibitory effects, are thought to inhibit bone resorption by inducing Fas ligands involved in apoptosis through estrogen receptors present in osteoclasts and reducing osteoclast life span (Nakamura et al., 2007; Tadaishi et al., 2014). GLP‐1 (glucagon‐like peptide‐1), a gastrointestinal hormone, is responsible for glycemic regulation functions, including incretin action and growth inhibition of pancreatic β‐cells, but it has also been reported in recent years that it is directly or indirectly involved in osteoblasts and osteoclasts (Hansen et al., 2018). Therefore, it is necessary to further examine the action mechanism of the bone metabolism improving effect such as the involvement in apoptosis and digestive tract hormone by MB intake.

In conclusion, continuous intake of MB in postmenopausal, healthy, Japanese, adult women improved the bone metabolic balance and may contribute to the maintenance of bone health, including the prevention of osteoporosis.

## CONFLICT OF INTEREST

Daiki Suehiro and Ken Fukami are employees of the San‐ei Sucrochemical Co., Ltd. San‐ei Sucrochemical Co., Ltd. supplied the maltobionic acid (MB) food product. Yuichiro Moriwaki, Sumiko Abe‐Dohmae (M.D.), and Motoko Ohnishi declare no conflicts of interest.

## AUTHOR CONTRIBUTIONS

**Daiki Suehiro:** Conceptualization (lead); Data curation (lead); Formal analysis (lead); Investigation (supporting); Methodology (equal); Resources (lead); Software (equal); Validation (equal); Visualization (lead); Writing – original draft (lead); Writing – review & editing (supporting). **Sumiko Abe‐Dhomae:** Writing – review & editing (supporting). **Yuichiro Moriwaki:** Investigation (lead). **Ken Fukami:** Conceptualization (supporting); Resources (supporting); Writing – original draft (supporting); Writing – review & editing (supporting). **Motoko Ohnishi:** Conceptualization (lead); Data curation (supporting); Formal analysis (supporting); Funding acquisition (equal); Investigation (equal); Methodology (equal); Project administration (equal); Supervision (equal); Validation (equal); Visualization (supporting); Writing – original draft (supporting); Writing – review & editing (lead).

## ETHICAL REVIEW

This study was approved by the Chubu University Certified Review Board on October 31, 2019 (no. 20190080).

## INFORMED CONSENT

Written informed consent was obtained from all study participants.

## Data Availability

The datasets used and/or analysed during the current study are available from the corresponding author on reasonable request.
